# The Impact of HIV on Maternal Morbidity in the Pre-HAART Era in Uganda

**DOI:** 10.1155/2012/508657

**Published:** 2011-10-12

**Authors:** Harriet Nuwagaba-Biribonwoha, Richard T. Mayon-White, Pius Okong, Peter Brocklehurst, Lucy M. Carpenter

**Affiliations:** ^1^ICAP, Mailman School of Public Health, Columbia University, P.O. Box 3567, Kampala, Uganda; ^2^Department of Primary Health Care, University of Oxford, Oxford, OX1 2-ET, UK; ^3^St. Raphael of St. Francis Hospital, Nsambya, Kampala, Uganda; ^4^Institute for Women's Health, University College London, London, WC1E AU, UK; ^5^Department of Public Health, University of Oxford, Oxford, OX3 7LF, UK

## Abstract

*Objective*. To compare maternal morbidity in HIV-infected and uninfected pregnant women. *Methods*. Major maternal morbidity (severe febrile illness, illnesses requiring hospital admissions, surgical revisions, or illnesses resulting in death) was measured prospectively in a cohort of HIV-infected and uninfected women followed from 36 weeks of pregnancy to 6 weeks after delivery. Odds ratios of major morbidity and associated factors were examined using logistic regression. *Results*. Major morbidity was observed in 46/129 (36%) and 104/390 (27%) of the HIV-infected and HIV-uninfected women, respectively, who remained in followup. In the multivariable analysis, major morbidity was independently associated with HIV infection, adjusted odds ratio (AOR) 1.7 (1.1 to 2.7), nulliparity (AOR 2.0 (1.3 to 3.0)), and lack of, or minimal, formal education (AOR 2.1 (1.1 to 3.8)). *Conclusions*. HIV was associated with a 70% increase in the odds of major maternal morbidity in these Ugandan mothers.

## 1. Introduction

Maternal morbidity is defined as illness in a woman who was or is pregnant from any cause related to the pregnancy, abortion, or child birth, excluding incidental or accidental causes [1]. In Uganda, the true level of maternal morbidity is unknown, but national estimates suggest that 5.1 maternal deaths occur for every 1,000 live births [2]. Although there has been a decline in maternal mortality since 2000 [3], child bearing is still associated with significant morbidity and mortality in this country. Like most sub-Saharan countries, Uganda has experienced a heavy burden of HIV-related disease in the last few decades. In men and non-pregnant women, HIV has been associated with at least a 2-fold increase in morbidity [4, 5] and 10–20-fold increase in mortality [6, 7]. Less is known about the association between HIV and maternal morbidity. We undertook a prospective observational study to describe, estimate, and compare the risk of maternal morbidity in HIV-infected and uninfected women in Uganda.

## 2. Methods

### 2.1. Setting

The study was conducted at St. Raphael of St. Francis Hospital Nsambya, in Kampala, Uganda, between November 2002 and November 2003. The hospital is a catholic missionary hospital located in the capital city (Kampala). During the study year, there were a total of 24,461 antenatal visits (of whom 6,016 were new bookings) and 10,768 deliveries recorded. The hospital provided care to general and private patients who paid at total of about $10 (general) and $40 (private) for antenatal care and a normal vaginal delivery. This hospital was one of the pilot sites for the Ugandan national programme to prevent mother-to-child HIV transmission (PMTCT) and the main source of antenatal, intrapartum and postnatal care for the local population [8]. During the study period all women attending the hospital for antenatal care were offered free opt-in HIV testing and counselling. Women were classified as HIV-infected if they had two positive rapid tests. HIV-infected women were given modified intrapartum obstetric care by delaying the rupture of membranes and avoiding episiotomies when possible. The main PMTCT regimen offered to women at the time was zidovudine monotherapy from 36 weeks of pregnancy to 1 week after delivery or a single dose of nevirapine given to HIV-infected women at the onset of labour. The use of highly active antiretroviral therapy (HAART) in pregnancy was not yet widely implemented. Exclusive breastfeeding was encouraged for both HIV-infected and uninfected mothers, but infected mothers could also give replacement feeds if after assessment this mode of feeding was deemed feasible.

### 2.2. Study Design

Pregnant women with known HIV status and willing to participate in the study were recruited into a prospective cohort at about 36 weeks of pregnancy and followed up until six weeks after the delivery. As maternal morbidity is known to be associated with parity [9], the cohort was restricted to nulliparous (para 0) and uniparous (para 1) women. HIV-infected and uninfected mothers were recruited in the ratio of 1 to 3. The main exposure of interest was the woman's HIV status and the primary outcome was any observed or self-reported major maternal morbidity. A woman was classified as having major maternal morbidity if she experienced one or more of the following: illness leading to hospital admission, severe febrile illness (fever lasting 3 days or longer), surgical revisions of abdominal or perineal wounds due to wound breakdown, and any illnesses that resulted in death. These maternal conditions have been classified as major morbidity by other authors [10–12]. 

Baseline data including demographic characteristics (nationality, residence, and age), socioeconomic confounders (education, marital status, and employment), medical history, and obstetric history were collected by interview in the antenatal clinic at 36–40 weeks of pregnancy using a semistructured questionnaire. CD4 tests were offered to HIV-infected women at baseline and analysed using routine standard laboratory procedures. Delivery data were abstracted from hospital records. A second interview was held six weeks after delivery for women to report on their morbidity during the study period. Women who did not attend the hospital for delivery and/or the 6 weeks postpartum appointment were telephoned and/or visited at home immediately after the missed hospital appointment.

The data were analysed using SPSS 11.5. Characteristics of HIV-infected and uninfected women were compared using either binary measures (percentages or odds ratios) where statistical significance of differences was examined with chi-squared test statistics, or means where differences were compared using *F* or *t* test statistics. In addition, CD4 measures were summarised using medians and interquartile ranges (IQR). Fisher's exact test was used when expected values in contingency table cells were less than 5, and Yates' correction was applied to chi-square tests on 2 × 2 tables. Odds ratios were first estimated crudely, without adjustment for potential confounders and then with adjustment for confounders (adjusted odds ratios, AOR) using logistic regression models. 

The study was approved in the UK by the Oxfordshire Tropical Research Ethics Committee (OXTREC) and in Uganda by the National AIDS Research Ethics Committee, the National Council of Science and Technology, and St. Raphael of St. Francis Hospital Nsambya administration.

## 3. Results

The characteristics of the 132 HIV-infected women and 399 HIV-uninfected women recruited into the cohort over the 12 month study period are summarized in Table 1. There were statistically significant differences between HIV-infected and uninfected women in parity, mean gestational age at recruitment, education, and infant feeding practices (Table 1). The majority of the HIV-infected women (100/132, 76%) were classified as asymptomatic (WHO stage 1). In 106 (80%) of HIV-infected women for whom CD4 counts at 36–40 weeks of pregnancy were available, the median CD4 count was 350/uL, (IQR 221 to 552/uL); 71 (67%) of the women had CD4 count >200/uL. PMTCT regimen data were available for 125 HIV infected women and the majority took monotherapy with AZT 74 (59%) or Nevirapine 48 (38.4%). Only 3 (2.4%) were on HAART and excluding them from the analysis had no significant impact on the results. 

At 6 weeks after delivery, follow-up data was available for 129/132 (97%) HIV-infected and 389/399 (97%) HIV uninfected women. Of these women, 46/129 (36%) who were HIV-infected, and 104/390 (27%) who were uninfected reported major morbidity. The crude odds ratio for any major morbidity in HIV-infected women compared to uninfected women was 1.52 (95% confidence interval 0.97 to 2.38, *P* = 0.05). The conditions that contributed to major morbidity are shown in Table 2. Two of the 6 hospital admissions among HIV-infected women occurred before delivery and were due to severe malaria. The remaining four admissions occurring after delivery were reported as due to severe malaria (2), wound sepsis and disruption (1) and a high fever (1). Eight of the 11 HIV-uninfected women were admitted to hospital before delivery with severe malaria (2), false labour (2), preeclampsia (1), urinary tract infection (1), diarrhoea (1), and psychosis (1). The three admissions after delivery in these women were due to severe malaria (1), wound sepsis and disruption (1), and postpartum haemorrhage (1). 

Severe febrile illness was the commonest contributor to major morbidity (Table 2), and was experienced by 34% (44/129) and 24% (93/390) of the HIV infected and uninfected women respectively. It occurred more often after delivery in 89% (39/44) of the HIV-infected women and 84% (78/93) of uninfected women. The crude odds ratio for severe febrile illness after delivery among HIV-infected women compared to uninfected women was 1.65 (95% confidence interval 1.05–2.60, *P* = 0.02). Severe febrile illness was less common in women who had Caesarean section deliveries (20/104, 19%) than those delivered vaginally (117/381, 31%) *P* = 0.03, and in women who were not exclusively breastfeeding (15/100, 15%) than in those breastfeeding (96/370, 26%), *P* = 0.03. Three HIV-infected women and 6 uninfected women had surgical revisions of abdominal incisions; one uninfected woman had an episiotomy resutured. 

Three deaths occurred after delivery, two of which were in HIV-infected mothers where the cause appeared AIDS related: one with pneumonia and the other with a high fever combined with vomiting and severe oral ulceration. The one death of an HIV-uninfected mother was a primigravidae woman who died from eclampsia and severe anaemia after delivering twins. Unadjusted analyses of each potential confounding factor revealed higher odds for any major morbidity among nulliparous women and women with no, or minimal, formal education while women aged 25–29 years seemed less likely to have major morbidity (not shown). 

When potential confounders (age, parity, private patients versus general patients, employment status, and education) were adjusted for, HIV status remained significantly associated with major morbidity among women at 6 weeks after delivery (Table 3), the adjusted odds ratio was 1.7 (95% CI 1.1 to 2.7), *P* = 0.02. Nulliparity (*P* = 0.003) and lack of or minimal, formal education (*P* = 0.02) remained significantly associated with higher odds for major morbidity in these adjusted analyses. 

Baseline CD4 counts were available for 103 (80%) of the 129 HIV-infected women who remained in follow-up. HIV-infected women who had major morbidity had significantly lower CD4 counts than those that did not have any major morbidity. The mean baseline CD4 count for the former group was 312/uL (standard deviation 177/uL) while in the latter group mean CD4 count was 456/ul (standard deviation 245/uL), *P* = 0.002. Major morbidity among HIV-infected women stratified by WHO HIV clinical stage at recruitment was 29/97 (30%) among women in WHO clinical stage I, 7/15 (58%) among women in WHO clinical stage II, and 10/16 (63%) among women in WHO clinical stage III, (chi-square test for linear trend = 7.9, *df* = 1, *P* = 0.005).

## 4. Discussion

This cohort study estimated the odds of any major morbidity to be 70% higher among HIV-infected women in Uganda. The commonest major morbidity was severe febrile illness, which occurred in 34% of HIV-infected and 24% of uninfected women. These results are in line with what has been found in other sub-Saharan African countries on the impact of HIV on major maternal morbidity leading to mortality [13, 14]. A five year audit in South Africa showed that maternal mortality (meaning women experienced major morbidity) was 6-times higher among HIV-infected women than infected women [15]. In Mozambique and Malawi, HIV-related illness was the leading cause of death among women who did not die of obstetric complications [16, 17]. HIV can aggravate other prevalent debilitating conditions in pregnancy like anaemia, thereby increasing severity of maternal morbidity [18, 19]. Similar findings have been observed in developed countries with HIV-infected pregnant women having a higher risk of hospitalisation, and longer stay during hospitalisation than HIV-uninfected women despite the use of HAART [20, 21]. Our study quantified the likely magnitude of the deleterious effect of HIV on maternal morbidity in Uganda, highlighting the need for interventions to address maternal morbidity as PMTCT programs scale up, even after HAART is widely available [21, 22]. In this cohort study, HIV-infected women delivered by Caesarean section had fewer severe febrile illnesses after delivery than those delivered vaginally. This finding is contrary to what has been observed by others [23–25], but in our study women who delivered by Caesarean section routinely had antibiotic prophylaxis while those who delivered vaginally did not. 

The strengths of this study lay in its prospective nature and in having a comparison group of women who were not infected with HIV. The same set of structured questions were used for both HIV-infected and uninfected women to limit biased inquiry about and reporting of maternal morbidity because HIV status of the mothers was generally known to the interviewers. Some limitations of the study should be noted: hospital admissions could overestimate major morbidity if women are admitted for minor conditions [26], but this was unlikely in our cohort where the financial and social costs of inpatient care are generally a deterrent to admissions in this setting. Laboratory investigations were limited in this study which meant that the study relied on clinical features and maternal reports. However, the results are applicable to resource-limited settings where many patients are managed similarly. Finally, this study was not designed or powered to examine maternal morbidity by ART status or HIV disease stage, but the stratified analysis implied higher major maternal morbidity with more advanced HIV disease. Further studies are needed to provide data on this as the combination of maternal morbidity and HIV-related morbidity can lead to a significantly negative impact on maternal health and survival. This study was limited to an urban missionary hospital in the capital city so future studies should include rural and government health facilities where the impact of HIV on maternal morbidity could be higher.

## 5. Conclusion

HIV infection increased the odds of major maternal morbidity by 70% in this cohort of Ugandan mothers giving birth for the first or second time. Maternal morbidity remains a significant health issue in such resource-limited settings and there is need for more interventions to address the effects of HIV infection on maternal morbidity.

## Figures and Tables

**Table 1:** Characteristics of HIV-infected and uninfected women in the cohort study.

	HIV infected *N* = 132 (% or SD)	HIV uninfected *N* = 399 (% or SD)	*P* value
*Sociodemographic characteristics*			
Private patients	16 (12)	46 (12)	1.0
Mean maternal age (standard deviation (SD))	24.9 (3.8)	23.5 (3.7)	0.002
Nulliparous	56 (42)	250 (63)	<0.001
4 or more antenatal visits in this pregnancy	94 (71)	273 (68)	0.6
Mean gestational age in weeks at recruitment (standard deviation)	36.1 (1.9)	36.7 (1.3)	<0.001
Highest level of formal education			
Primary school or no formal education	36 (27)	79 (20)	
Secondary school	58 (44)	165 (41)	0.02
Tertiary (higher) education	38 (29)	155 (39)	
Women without own income	75 (57)	233 (58)	0.9
Married or cohabiting	102 (77)	341 (86)	0.04
Single	32 (23)	59 (14)	
Place of delivery			
St. Francis Hospital*	115 (87)	344 (86)	1.0
Home or other health care setting	16 (13)	50 (12)	
Mode of delivery*			
Vaginal delivery	88 (77)	251 (79)	
Elective Caesarean section	6 (5)	7 (2)	0.2
Emergency Caesarean section	20 (18)	65 (19)	
Infant feeding practices 6 weeks after delivery**			
Exclusive breast feeding	75 (63)	299 (80)	
Replacement feeding	39 (32)	8 (2)	<0.001
Mixed feeding	6 (5)	68 (18)	

*Mode of delivery data only available for 459 births that occurred at St. Francis Hospital.

**Infant feeding practices data only available for 120 and 375 births.

**Table 2:** Major morbidity in HIV-infected and uninfected women by 6 weeks after delivery.

	HIV infected *N* = 129 (%)	HIV uninfected *N* = 390 (%)	Crude odds ratio (95% CI)	*P* value
Hospital admissions	6 (5)	11 (4)	1.68 (0.54–5.04)	0.31
Severe febrile illness	44 (34)	93 (24)	1.65 (1.05–2.60)	0.02
Surgical revision	3 (2)	7 (2)	1.30 (0.26–5.68)	0.71*
Illnesses causing death	2 (2)	1 (0)	6.13 (0.43–172.02)	0.15*

Major morbidity (women with any of the above)	46 (36)	104 (27)	1.52 (0.97–2.38)	0.05

*Fisher's exact 2-tailed *P* value.

**Table 3:** Major morbidity due to HIV infection adjusted for selected demographic factors.

	*N* = 519	Adjusted odds ratio (95% CI)	*P* value
HIV infection	129	1.7 (1.1–2.7)	0.02
Nulliparity	299	2.0 (1.3–3.0)	0.003
Private patients	60	0.9 (0.4–1.7)	0.64
Women without own income	300	0.8 (0.5–1.3)	0.33
Education			
Tertiary education	110	Reference	—
Secondary education	220	1.9 (1.1–3.1)	0.02
Primary or no education	189	2.1 (1.1–3.8)	0.02
Age			
Age <20 years	68	Reference	—
Age 20–24	236	1.1 (0.6–2.0)	0.77
Age 25–29	178	0.7 (0.4–1.5)	0.43
Age 30+	37	0.7 (0.3–2.1)	0.57
